# Dosimetric comparison of surface mould HDR brachytherapy with VMAT

**DOI:** 10.1002/jmrs.301

**Published:** 2018-08-13

**Authors:** Eeva L. Boman, Dean B. Paterson, Shelley Pearson, Nichola Naidoo, Carol Johnson

**Affiliations:** ^1^ Blood & Cancer Centre Wellington Hospital Wellington New Zealand; ^2^ Department of Oncology Tampere University Hospital Tampere Finland; ^3^ Department of Medical Physics Tampere University Hospital Tampere Finland

**Keywords:** Brachytherapy, Freiburg Flap, Intensity‐modulated radiation therapy, scalp, skin, volumetric‐modulated arc therapy

## Abstract

**Introduction:**

The aim of this study was to investigate the dosimetric differences between surface mould high‐dose‐rate (HDR) brachytherapy and external beam volumetric‐modulated arc therapy (VMAT) for two treatment sites.

**Methods:**

Previously treated HDR brachytherapy surface mould scalp (*n* = 4) and lower leg (*n* = 3) treatments were retrospectively analysed. The VMAT plans were optimised using an additional 3‐mm setup margin on the clinical target volume (CTV) of the previously treated HDR plans. The HDR plans were calculated and normalised using the TG‐43 formalism and recalculated with Acuros BV (AC).

**Results:**

On average, the mean brain and normal tissue doses were reduced by 44.8% and 27.4% for scalp and lower leg VMAT cases, respectively, when compared to AC calculated HDR plans. For VMAT plans, the average dose to a 1‐mm thick skin structure deep to the target volume was not any lower than that in AC HDR plans. On average, the CTV coverage was 13.8% and 9.6% lower for scalp cases with AC dose calculation than with TG‐43 and 8.3% and 5.3% lower for lower leg cases if 0‐ or 1‐cm backscatter material was applied above the catheters, respectively.

**Conclusions:**

VMAT is a feasible treatment option in the case of extensive skin malignancies of the scalp and lower leg. Uncertainties related to delivered dose with HDR brachytherapy when using the TG‐43 dose calculation model or possible air gaps between the mould and skin favour the use of VMAT. The potential soft tissue deformation needs to be considered if VMAT is used.

## Introduction

High‐dose‐rate (HDR) brachytherapy is a common treatment option for skin malignancies.^1^, ^2^ The use of shielded cup‐shaped applicators is limited to lesions of less than 3 cm in diameter.^3^ Skin lesions larger than 3 cm are defined as extensive skin lesions. At our institution, surface mould brachytherapy is considered for patients with wide spread areas of in‐transit melanoma metastases that cannot be easily surgically excised. Available treatment modalities for these extensive skin lesions are HDR brachytherapy,^1^, ^2^ external megavoltage electron beams,^4^ intensity‐modulated external megavoltage photon beams,^5^, ^6^, ^7^, ^8^ and electronic brachytherapy.^9^ A number of reports have demonstrated that intensity‐modulated techniques have reduced organ at risk (OAR) dose and increased dose homogeneity to the treatment volume when compared to HDR brachytherapy and/or megavoltage electron treatments with multiple field junctions.^5^, ^6^, ^7^, ^8^

The TG‐43 formalism is widely used for dose calculation in clinical brachytherapy practice.^10^ TG‐43 assumes full scatter in water and does not take into account the actual scatter conditions of the patient or the surrounding environment.^3^ Recently, model‐based dose calculation algorithms (MBDCA) such as Acuros BV (Acuros BV^TM^, Varian Medical Systems, Palo Alto, CA) and ACE (Elekta, Stockholm, Sweden) have become available in most clinical treatment planning systems.^11^ In contrast to the TG‐43 dosimetry formalism, these algorithms take heterogeneities and the actual scatter conditions into account and calculate either dose delivered to water or dose delivered to the actual medium. Both Acuros BV and ACE have been shown to agree within ±2% with Monte Carlo method calculations for single‐source models,^12^ and specifically near the skin for breast brachytherapy patient models.^13^ We have recently shown that the delivered dose can be up to 15% lower at the prescription depth than that seen in the TG‐43 model for surface mould HDR brachytherapy and the difference increased with the skin lesion size (treatment area).^14^ This might not be a significant issue when MBDCAs are accepted in clinical use.^11^ However, in the current clinical practice, this uncertainty in HDR brachytherapy dose calculation is much larger than that seen in external beam dose calculations.

The primary aim of this study is to investigate whether volumetric‐modulated arc therapy (VMAT) is a feasible treatment option for extensive skin malignancies of the scalp or lower leg in comparison to surface mould Ir‐192 HDR brachytherapy. In addition, a secondary aim was to compare the dosimetric differences between TG‐43 and the Acuros BV MBDCA for the two treatment sites. These differences are studied in clinical cases with and without backscatter material applied above the treatment catheters.

## Methods

Patients previously treated at the Wellington Blood & Cancer Centre between 2015 and 2016 with surface mould HDR brachytherapy for extensive melanoma of the scalp (*n* = 4) and lower leg (*n* = 3) were included in this retrospective study, which was conducted in 2017. All patients gave consent for use of their data for clinical audit. Locality approval was granted by the study institution's Research Office. This study was considered routine practice development and ‘out of scope’ confirmation was acquired from the New Zealand Health and Disability Ethics Committee. The CT data sets were anonymised and exported to the institution's testing and development planning system. Patient anonymity was guaranteed in the study.

**Figure 1:**
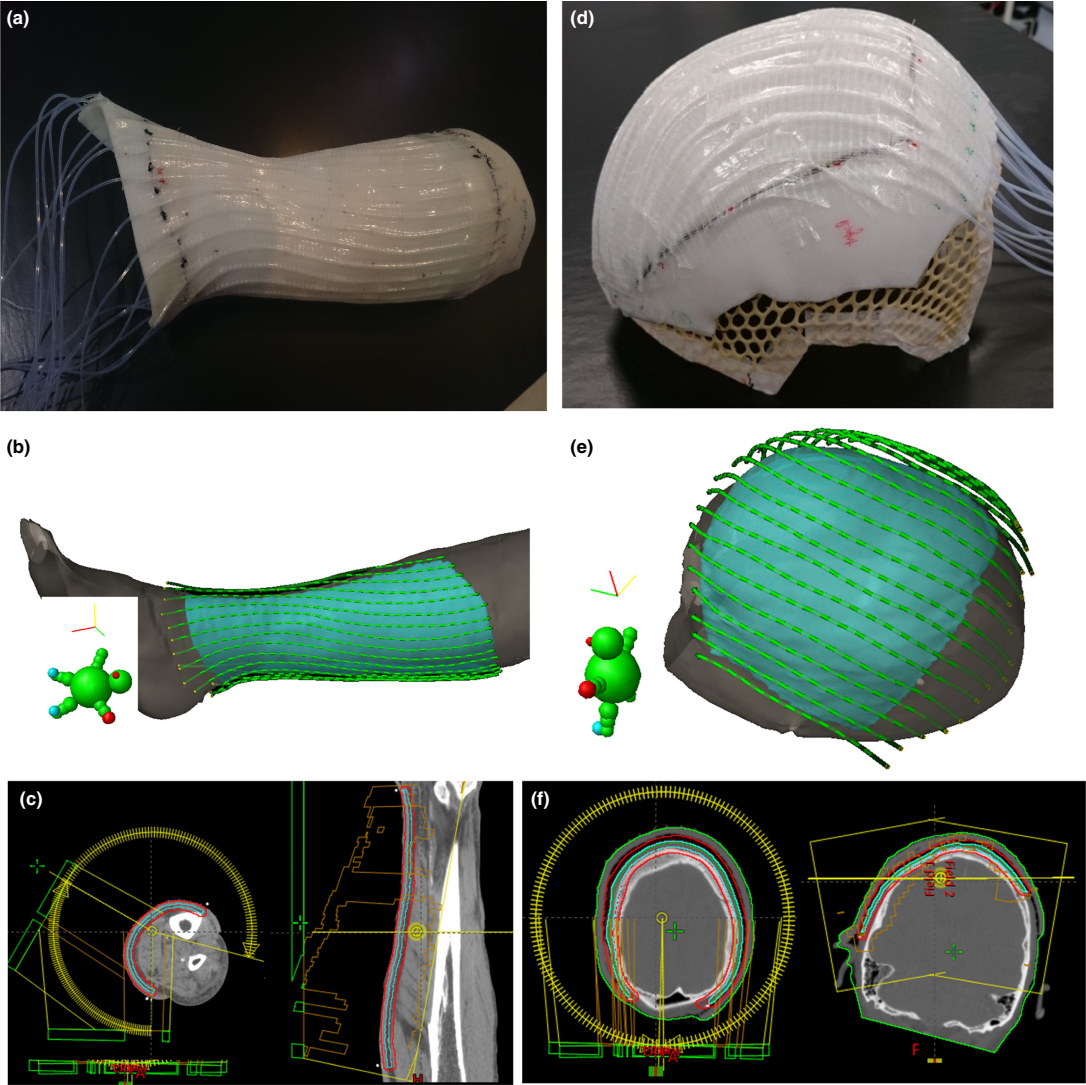
Examples of lower leg (L3) (A, B, C) and scalp (S1) (D, E, F) cases. The high‐dose‐rate brachytherapy mould with catheters attached (A and D), brachytherapy planning view of the clinical target volume (CTV) (cyan) with source positions (B and E) and VMAT gantry rotations with PTV (red) and CTV (cyan) (C and F).

The patients were treated using surface mould HDR brachytherapy with an ^192^Ir source (VS2000, Varian Medical Systems). All scalp and two lower leg cases had a custom made mould, which consisted of 4.8 mm Aquaplast RT Custom Bolus (Qfix Avondale, PA) attached to 2.4 mm Fibreplast Slimline (Qfix). 4.7Fr (1Fr (French gauge) = 0.033 cm) plastic catheters (Varian Medical Systems) were taped over the mould with approximately 1.0 cm spacing (see Figure 1A/D). For one lower leg case, a Freiburg Flap (FF) (Elekta) mould was used. In all cases, the distance from the catheter to skin surface was ~ 5 mm in order to reduce the dose inhomogeneity at the skin surface.^3^ CT imaging (Philips Brilliance Big Bore, Philips, the Netherlands) was performed with the mould in place as part of treatment preparation using a transversal slice thickness of 1.5 mm. No additional backscatter material was used above the moulds. For the previously treated HDR brachytherapy plans (PHDR_TG‐43_), dwell time optimisation was based on the TG‐43 dose calculation algorithm with a 0.1 cm grid size in Brachytherapy Planning version 13.7 (v13.7) (Varian Medical Systems). For comparison, the dose to water (transport in medium) was also calculated using Acuros BV v13.7 with a 0.1 cm grid size (PHDR_AC_). The clinical target volume (CTV) was marked on the skin by the radiation oncologist with a 1–2 cm margin on the lesions and a treatment depth of 3 mm. Table 1 lists the CTV size and HDR brachytherapy source characteristics for each case. The prescribed dose was 30 Gy in five fractions with two fractions per week. PHDR_TG‐43_ plans were normalised such that 95% and 90% of the CTV volume received 100% of the prescription dose for the scalp (D95_CTV_S_ = 100%) and lower leg (D90_CTV_L_ = 100%) lesions, respectively. PHDR_AC_ had the same source positions and dwell times as PHDR_TG‐43_. The plans were also recalculated using Acuros BV with 1 cm water equivalent bolus simulated above the catheters (PHDR_AC+1_).

**Table 1:** CTV and PTV volume (V_CTV_ and V_PTV_, respectively) and CTV area on the skin with high‐dose‐rate (HDR) catheter details (number of catheters (#), average loading length per catheter and step size) for each scalp (S1, S2, S3, S4) and lower leg (L1, L2, L3) case

	V_CTV_ (cm^3^)	V_PTV_ (cm^3^)	CTV Area (cm^2^)	# HDR catheters	Average loading length (min–max) (cm)	Step size (cm)
Scalp1 (S1)	88.2	276.1	294.0	18	17 (9–21)	1.0
Scalp2 (S2)	102.7	317.4	342.3	18	28 (18–32)	1.5
Scalp3 (S3)	167.4	488.0	558.0	20	20 (8–26)	0.5/1.0
Scalp4 (S4)	187.5	546.6	625.0	23	26 (5–36)	1.0
Leg1 (L1)^a^	44.0	144.4	146.7	15	12 (5–14)	1.0
Leg2 (L2)	93.1	306.1	310.3	13	24 (13–26)	1.0
Leg3 (L3)	116.6	357.8	388.7	18	25 (10–29)	1.0

a Freiburg flap.

For VMAT plans (PVMAT), the same CT data sets were used and retrospective plans were created in External Beam Planning (Varian Medical Systems) using the Analytic Anisotropic Algorithm (AAA) v13.7 dose calculation algorithm, a 0.1 cm grid size and Millennium 120 MLC. All plans were designed to be delivered within TrueBeam linear accelerator (Varian Medical systems) limitations. The HDR brachytherapy catheters, patient immobilisation devices and treatment couch were excluded from the calculations. The bolus material (4.8 mm Aquaplast RT Custom Bolus (Qfix)) of the surface mould was used as bolus in the PVMAT plans. The planning target volume (PTV) was created from the CTV with a 3‐mm expansion.^15^ The PTV volumes are shown in Table 1. Scalp PVMAT plans consisted of two full arcs with collimator rotations of 80° and 110°. A maximum of 15° couch rotation was used if the lesion was located more on the lateral side of the scalp. The lower leg PVMAT plans consisted of two full arcs with collimator rotations of 10° and 350° and included a 75° avoidance sector to avoid irradiation through the contralateral leg. All PVMAT plans were normalised to D98%_PTV_ = 95% of the prescription dose (30 Gy in five fractions).

Several structures were created for dosimetric comparison: for all cases, a 1 mm skin surface structure was created from the CTV (CTV1mm) (D95_CTV1mm_ (%), mean _CTV1mm_ (%)); for the scalp cases, brain (mean_BRAIN_ (Gy), D50_BRAIN_ (%), D0.1cc_BRAIN_ (Gy)) and lenses (D0.1cc_LENS_L_ (Gy), D0.1cc_LENS_R_ (Gy); and normal tissue (NT) for the lower leg cases (mean_NT_ (Gy), D50_NT_ (%), D0.1cc_NT_ (Gy)). The NT structure was created by cropping the ‘BODY’ structure 13 mm from the CTV. Maximum doses were investigated for CTV and PTV (D0.1cc_CTV_ (Gy) and D0.1cc_PTV_ (Gy)).

To evaluate target coverage and plan quality, the Paddick conformity index (CI)^16^ was calculated as;

(1) $$\text{CI}=\frac{{\text{VX}}_{\mathrm{TV}}}{V_{\mathrm{TV}}}\frac{{\text{VX}}_{\mathrm{TV}}}{\text{VX}}$$

in which, VX%_TV_ (cc) is the volume which receives at least X% of the prescribed dose in the target volume (TV), V_TV_(cc) is the TV volume and VX(cc) is the volume which receives at least X% of the prescribed dose. CI = 1 indicates the optimal plan quality whereas lower values indicate a poorer plan quality. The CTV was used as the target volumes for HDR brachytherapy (X was 95% and 90% for scalp and lower leg cases, respectively) whereas the PTV was used for PVMAT plans (X was 95%).

## Results

### For scalp cases

**Figure 2:**
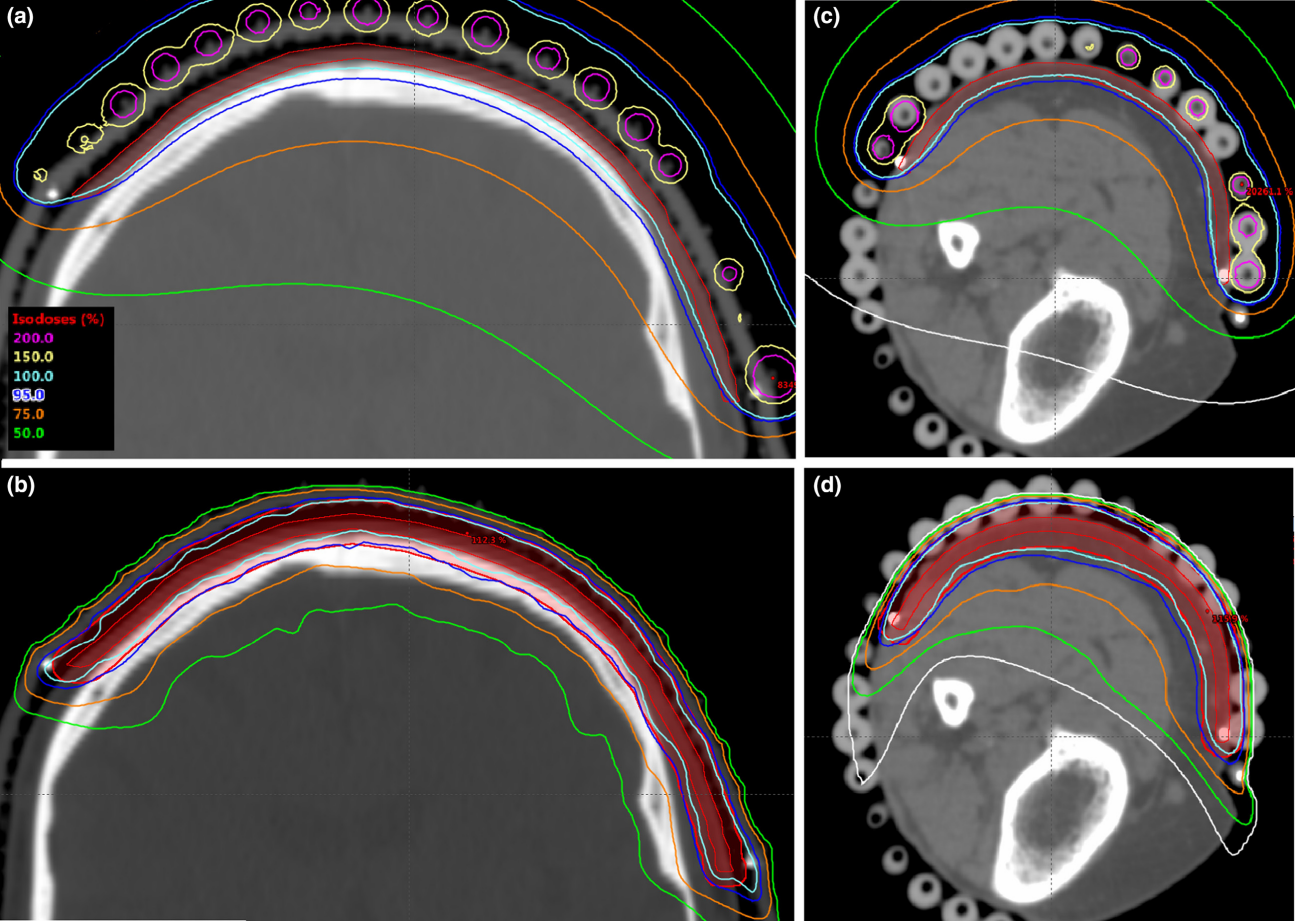
Dose distributions for PHDR_TG__‐43_ (A and C) and PVMAT (B and D) plans for the scalp (coronal view for case S1) (A and B) and lower leg (transversal view for L1 case) (C and D) cases. For all cases, the clinical target volume (CTV) is shown in red colour. In addition, for VMAT plans, the PTV is indicated with the same colour as the CTV. The isodose lines are shown in a subfigure A (100% isodose indicates the prescription dose).

The CTV size ranged from 294 to 625 cm^2^ covering approximately one‐third (S1 and S2), half (S3) or the whole scalp (S4). Figure 2(A and B) shows the dose distributions of PHDR_TG‐43_ and PVMAT plans for one scalp case (S1) in the coronal plane. The dosimetric comparison of the four scalp cases between PHDR_TG‐43_, PHDR_AC_, PHDR_AC+1_ and PVMAT plans are shown in Table 2. The CTV coverage decreased more than 13% (D95_CTV_ ≤ 86.5%) and 10% (D95_CTV_ ≤ 90.8%) for PHDR_AC_ and PHDR_AC+1_, respectively, in all cases when compared to PHDR_TG‐43_ plans. Thus, the addition of 1 cm backscatter material reduced the dose difference by 3%. All PVMAT plans were superior in terms of CI and OAR doses when compared to HDR brachytherapy plans. The near maximum doses in the CTV were highest in the PHDR_TG‐43_ plans (on average (±SD) D0.1cc_CTV_ = 129.1 ± 9.2%). The near maximum dose for PHDR_AC_ plans (D0.1cc_CTV_ = 118.3 ± 7.8%) were closer to that of the PVMAT plans (D0.1cc_PTV_ = 112.1 ± 2.3%). The treatment times and the number of monitor units (MUs) are presented in Table 3. The average (±1SD) treatment times were 31.4 ± 2.9 min for HDR brachytherapy plans and 5.5 ± 0.5 min for VMAT plans.

**Table 2:** Dose parameters for CTV/PTV and OARs for high‐dose‐rate (HDR) brachytherapy plans (PHDR_TG‐43_ and PHDR_AC_, PHDR_AC+1_) and VMAT plans (PVMAT) for four scalp cases (S1, S2, S3 and S4). PTV results are not presented for HDR plans

	D95_CTV_ (%)	D95_CTV1mm_ (%)	CI_CTV,PTV_ a	D0.1cc_CTV,PTV_ a (%)	Mean_CTV1mm_ (%)	Mean_BRAIN_ (Gy)	D0.1cc_BRAIN_ (Gy)	D0.1cc_LENS_L_ (Gy)	D0.1cc_LENS_R_ (Gy)
S1									
PHDR_TG‐43_	100.0	104.9	0.64	125.8	110.5	11.9	26.9	14.0	11.6
PHDR_AC_	86.2	91.0	0.28	114.3	97.2	9.9	22.6	11.3	9.0
PHDR_AC+1_	90.5	95.8	0.64	118.7	102.0	10.5	23.8	12.1	9.8
PVMAT	102.8	103.4	0.87	115.5	106.8	4.5	27.2	10.3	4.4
S2									
PHDR_TG‐43_	100.0	106.1	0.56	123.2	112.4	12.5	27.1	15.7	11.7
PHDR_AC_	86.5	92.2	0.45	111.5	98.2	10.4	22.4	13.0	9.4
PHDR_AC+1_	90.8	97.2	0.76	116.5	103.2	10.9	23.5	13.7	10.0
PVMAT	100.9	101.4	0.90	111.1	103.7	6.0	26.7	11.1	3.4
S3									
PHDR_TG‐43_	100.0	106.9	0.49	122.5	112.4	14.6	26.9	12.5	9.9
PHDR_AC_	86.1	91.4	0.45	115.9	96.8	12.2	22.2	10.0	7.5
PHDR_AC+1_	90.0	96.2	0.72	119.5	101.4	12.8	23.3	10.3	7.9
PVMAT	99.9	100.6	0.76	109.3	103.1	8.5	24.4	3.7	1.7
S4									
PHDR_TG‐43_	100.0	104.3	0.49	144.8	114.4	18.4	29.2	15.8	11.7
PHDR_AC_	86.0	88.8	0.45	131.6	99.7	15.7	25.1	12.9	9.2
PHDR_AC+1_	90.7	93.9	0.63	135.9	103.3	16.5	26.3	13.6	9.9
PVMAT	97.6	96.9	0.79	112.3	102.4	7.7	28.0	14.7	7.1

a CTV is used for HDR plans and PTV for VMAT plans.

**Table 3:** Dose parameters for CTV/PTV and OARs for high‐dose‐rate (HDR) brachytherapy plans (PHDR_TG‐43_ and PHDR_AC_, PHDR_AC+1_) and VMAT plans (PVMAT) for three lower leg cases (L1, L2 and L3). PTV results are not presented for HDR plans

	D90_CTV_ (%)	D90_CTV1mm_ (%)	CI_CTV,PTV_ a	D0.1cc_CTV,PTV_ a (%)	Mean_CTV1mm_ (%)	D0.1cc_NT_ (Gy)	D50_NT_ (Gy)	Mean_NT_ (Gy)
L1								
PHDR_TG‐43_	100.0	107.0	0.70	144.4	114.5	20.8	8.9	9.4
PHDR_AC_	92.6	99.2	0.70	137.8	107.0	18.7	7.5	8.0
PHDR_AC+1_	95.1	102.1	0.79	139.3	107.8	19.3	7.8	8.4
PVMAT	104.8	106.2	0.78	118.3	110.5	23.8	2.3	5.1
L2								
PHDR_TG‐43_	100.0	107.6	0.58	133.6	113.8	22.5	9.8	10.6
PHDR_AC_	94.2	101.5	0.76	127.2	107.7	20.8	8.2	9.1
PHDR_AC+1_	96.4	104.1	0.69	128.3	110.3	21.7	8.6	9.5
PVMAT	105.7	106.5	0.61	123.7	110.9	29.4	3.1	6.9
L3								
PHDR_TG‐43_	100.0	107.2	0.63	119.5	111.9	22.5	10.5	11.2
PHDR_AC_	88.4	94.9	0.54	114.0	100.1	19.5	8.4	9.0
PHDR_AC+1_	92.6	99.4	0.78	117.9	104.3	20.5	9.0	9.6
PVMAT	103.5	103.9	0.59	117.0	107.7	32.4	2.3	7.1

a CTV is used for HDR plans and PTV for VMAT plans.

### For lower leg cases

The CTV size ranged from 146.7 to 388.7 cm^2^, covering the approximate length and half the circumference of the lower leg. Figure 2(C and D) shows the dose distributions of PHDR_TG‐43_ and PVMAT plans for one case (L1) in the transversal plane. The dosimetric parameters for each lower leg case are shown in Table 4. The CTV coverage (D90%_CTV_) decreased by at least 5.8% for PHDR_AC_ when compared to PHDR_TG‐43_ plans. The addition of 1 cm backscatter material over the treatment catheters reduced the difference by ~ 2%. The near maximum doses were higher for PHDR_TG‐43_ (on average D0.1cc_CTV_ = 132.5 ± 10.2%) and PHDR_AC_ (126.4 ± 9.7%) than that for the PVMAT plans (D0.1cc_PTV_ = 119.6 ± 2.9%). However, the mean_CTV1mm_ dose was larger for PVMAT (109.7 ±1.4%) than that for PHDR_AC_ plans (104.9 ± 3.4%). The dose to the normal tissue was lower in all PVMAT plans when compared to HDR brachytherapy plans. The average treatment times were 21.2 ± 3.9 min and 6.2 ±0.9 min for HDR and VMAT treatments, respectively (Table 3).

**Table 4:** Irradiation time (min) for high‐dose‐rate (HDR) plans (PHDR_TG‐43_ and PHDR_AC_, PHDR_AC+1_) and VMAT plans (PVMAT) for all cases (S1‐4, L1‐3) with the number of monitor units (MU) for each VMAT plan

	S1	S2	S3	S4	L1	L2	L3
HDR time (min)^a^	25.3	26.6	37.7	36.1	12.6	23.5	27.2
VMAT time (min)	2.7	2.4	2.6	3.8	4.1	3.9	3.8
VMAT MU	1581	1453	1559	2285	2285	2326	2250

a Assuming 10Ci source.

## Discussion

We have shown that EBRT using VMAT is a feasible alternative to surface mould HDR brachytherapy in the treatment of extensive skin malignancies. Our results show that, in both cases (scalp and lower leg), the OAR doses were lower for VMAT plans when compared to HDR brachytherapy, even when dose is calculated using Acuros BV to accurately take into account the scatter conditions and tissue inhomogeneities. The near maximum CTV dose for PHDR_AC_ plans was similar to the near maximum dose for the PTV in VMAT plans for scalp cases. For lower leg cases, the near maximum CTV dose was slightly higher for PHDR_AC_ plans than that for PTV in PVMAT plans.

The mean and D95 doses for the CTV1mm surface volume were relatively similar amongst TG‐43 calculated HDR brachytherapy plans and VMAT plans. These results should be interpreted with caution because of the dimensions of the volume (1 mm thickness) and the dose calculation grid (1 mm). However, we believe that the magnitude of dose is correct and aligns with what was observed in the CTV.

We have previously demonstrated that the dose difference between TG‐43 and Acuros BV calculations increase with increasing loading area for HDR brachytherapy surface mould treatments, resulting in a lower actual delivered dose than that seen with the TG‐43 dose calculation model.^14^ The dose difference seen at the prescription depth for scalp treatments was up to 16%. In the same study, we showed that the presence of bone under the treatment area increased the difference seen between TG‐43 and Acuros BV calculations. In this study, the dose difference between the PHDR_TG‐43_ and PHDR_AC_ plans for lower leg cases were slightly less than that seen for scalp cases. The treatment areas were slightly smaller for lower leg cases than the scalp cases, which might explain the differences. In addition, for the scalp, the presence of bone might have more influence than that for the lower leg cases. One lower leg case (L1) had a FF mould, which is constructed in a different manner to the custom moulds; thus, the difference seen between TG‐43 and Acuros BV calculations might be different than those seen in the study of Boman et al.^14^ Currently, the American Brachytherapy Society (ABS) working group^3^ recommends not to use bolus as a backscatter material for skin HDR brachytherapy treatments. In this study, we have demonstrated in seven clinical examples that the addition of backscatter material above the treatment catheters not only reduced the dose difference between the Acuros BV and TG‐43 calculated D95CTV on average by 4.3% and 3.0% for scalp and lower leg cases, respectively, but also increasing the OAR dose. Clinicians should be aware of these dose differences and reflect upon them in the context of historical clinical outcomes when prescribing HDR brachytherapy surface mould treatments.

For 6 MV external photon beams, Butson et al.^17^ studied the skin dose under bolus with the presence of air gaps, for 10 × 20 cm^2^ treatment fields a maximum difference of 2% and 4% was seen in skin dose for 4 and 10 mm air gaps, respectively. In addition, Mahdavi et al.^18^ measured an approximate 9% surface dose decrease resulting from a 5 mm air gap under bolus for a 6 MV VMAT head and neck case with 3 or 5 mm bolus thickness. For HDR brachytherapy treatments, the presence of additional air gaps due to positional variation of the mould (from planned) would lead to underdosing the target volume as the distance from the source positions is increased when compared to planned positions. In our own simulations using Acuros BV dose calculations, the addition of a 4 or 10 mm air gap between the mould and the skin decreased the dose by approximately 67% and 87%, respectively, at the prescription depth of 5 mm under the skin. This clearly indicates that for HDR brachytherapy, the presence of air gaps under the mould has a greater dosimetric impact than air gaps under bolus in VMAT treatments.

In this study, we used a 3 mm CTV‐PTV margin for the EBRT treatments. It was assumed that these patients, if treated with EBRT, would have appropriate thermoplastic mask fixation. The 3‐mm margin was selected to compensate for possible patient inter‐ and/or intrafraction motion and image matching system errors. With the option of six degrees of freedom (6DoF) couch systems and CBCT image guidance, we think this margin selection was appropriate, at least for the scalp cases. A CBCT match for the scalp is relatively straightforward and any skin (PTV) deformation is minimal. For the lower leg cases, a more cautious approach is recommended if EBRT is considered. Soft tissue deformation might be an issue in the treatment setup as the skin lesion may deform and/or move relative to the bone anatomy and a larger margin should be considered. Furthermore, the maximum length of the CBCT scan may limit the match as the target volume may be longer than the maximum length of the CBCT.

Slightly different normalisation methods were used between VMAT and HDR plans. All VMAT cases were normalised to the PTV (D98%_PTV_ = 95%) and HDR plans were normalised to the CTV (D95_CTV_S_ = 100% and D90_CTV_L_ = 100%) with TG‐43 dose calculation as per standard practice in our institution. These different normalisation methods may confound the comparisons. However, the dose calculation differences seen between TG‐43 and AC, these normalisation differences between treatment modalities become irrelevant if the more accurate AC dose calculation model is used in the dose comparisons.

The FF applicator is relatively easy to use and easy to produce acceptable treatment plans in lower leg cases. In addition, the FF avoids the time and resources required to construct a custom mould. The limitation of the FF, for extensive scalp lesions, is its inability to conform to the spherical curvature of the scalp. In addition, tissue in the vicinity of a limb treatment area is more likely to deform in shape when compared to the scalp. A flexible surface mould applicator will deform with the limb; however, VMAT cannot easily adapt to this. For these reasons, HDR brachytherapy might be considered a favourable treatment option for limb cases.

Kai et al.^19^ investigated the delivery times between VMAT and intensity modulated RT (IMRT) for the treatment of scalp angiosarcoma and concluded that VMAT plan quality was comparable to a 9‐field IMRT plan with reduced delivery time. The treatment times are much longer for HDR brachytherapy treatments than those for VMAT or IMRT plans. Additionally, in our experience, the time required to construct a custom mould for HDR brachytherapy is much greater than the time required for construction of an EBRT fixation device. This may be an issue in terms of cost and for elderly and/or sick patients.

The case report of Santos et al.^7^ compared the dosimetric parameters for extensive scalp (frontal area) lesions for HDR and VMAT plans and found that the OAR doses were similar with both techniques. They introduced the concept of a tangential VMAT technique with multiple (five) half arcs, which reduced the OAR dose significantly. Although the four scalp cases investigated in this study included more extensive treatment areas than that used in the study of Santos et al.,^7^ the results are similar: the OAR dose is reduced by the VMAT technique. Additionally, the dose coverage is improved with the VMAT technique when compared to PHDR_AC_ or PHDR_AC+1_ plans, which are considered to present the actual dose in the patient more accurately than that in PHDR_TG‐43_ plans. Even the surface dose (mean_CTV1mm_) is similar for VMAT plans if compared to Acuros BV‐calculated plans. For these reasons, the use of VMAT is seen as a favourable treatment option in the treatment of extensive scalp lesions.

## Conclusion

Our study demonstrates that VMAT could be a feasible treatment option for skin malignancies instead of surface mould HDR brachytherapy. The uncertainties related to the HDR brachytherapy dose calculation model (TG‐43) should be acknowledged if HDR brachytherapy is used. The use of AC dose calculation model would reduce this uncertainty. In addition, the large dosimetric errors resulting from possible air gaps under the mould might favour the EBRT option. Careful consideration is needed in the cases such as limbs where soft tissue deformation may cause a problem.

## Conflict of Interest

The authors declare no conflict of interest.
